# Bioinformatics analysis of the locus for enterocyte effacement provides novel insights into type-III secretion

**DOI:** 10.1186/1471-2180-5-9

**Published:** 2005-03-09

**Authors:** Mark J Pallen, Scott A Beatson, Christopher M Bailey

**Affiliations:** 1 Bacterial Pathogenesis and Genomics Unit, Division of Immunity and Infection, Medical School, University of Birmingham, Birmingham, B15 2TT, UK

## Abstract

**Background:**

Like many other pathogens, enterohaemorrhagic and enteropathogenic strains of *Escherichia coli* employ a type-III secretion system to translocate bacterial effector proteins into host cells, where they then disrupt a range of cellular functions. This system is encoded by the locus for enterocyte effacement. Many of the genes within this locus have been assigned names and functions through homology with the better characterised Ysc-Yop system from *Yersinia* spp. However, the functions and homologies of many LEE genes remain obscure.

**Results:**

We have performed a fresh bioinformatics analysis of the LEE. Using PSI-BLAST we have been able to identify several novel homologies between LEE-encoded and Ysc-Yop-associated proteins: Orf2/YscE, Orf5/YscL, rORF8/EscI, SepQ/YscQ, SepL/YopN-TyeA, CesD2/LcrR. In addition, we highlight homology between EspA and flagellin, and report many new homologues of the chaperone CesT.

**Conclusion:**

We conclude that the vast majority of LEE-encoded proteins do indeed possess homologues and that homology data can be used in combination with experimental data to make fresh functional predictions.

## Background

Type-III secretion is one of five different types of protein secretion employed by Gram-negative bacteria to move proteins from the cytoplasm across two membranes to the external milieu [1-5]. Any given type-III secretion system (T3SS) consists of a multi-protein complex that spans both the inner and outer membranes and the periplasm so that proteins are delivered to the exterior in an ATPase-dependent fashion without a periplasmic intermediate. Type-III secretion systems can be classified into two major groups: those associated with flagellar biosynthesis and those associated with interactions between bacteria and eukaryotic cells [5]. Type-III secretion is thus central to our understanding of bacterial motility, virulence, symbiosis, and ecology. Type-III secretion also provides an attractive drug and vaccine target [6] and has been exploited in the biotechnology arena as a antigen delivery system [7,8]

**Figure 1:**
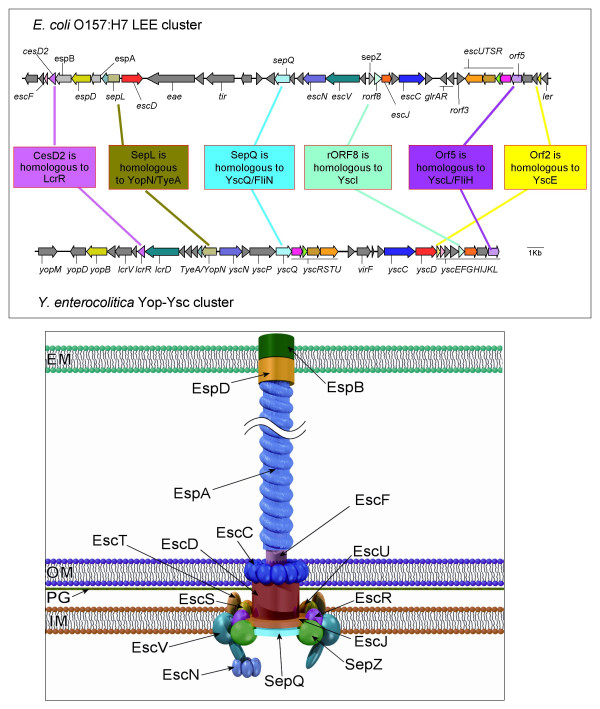
**1a: A comparison between the *E. coli *O157:H7 LEE and the *Yersinia enterocolitica *Yop-Ysc clusters. 1b: Graphical representation of the LEE-encoded type-III secretion system. **The LEE region between EspF and Ler is shown (4589000–4620000 of the *E. coli *O157:H7 RIMD 0509952 genome. Accession number: dbj|BA000007.2). The Yop-Ysc cluster between YopM and YscL is shown (15000–40000 of the *Yersinia enterocolitica *plasmid pYVe227 Accession number: dbj|BA000007.2gb|AF102990). Genes shaded in the same solid colour exhibit previously known homologies between the LEE and Ysc/Yop cluster. Genes shaded in the same colour but with horizontal hatching are homologues where we have added information in this paper. Genes which show no apparent homology to any the Ysc/Yop system, but which encode proteins that appear in our graphical representation are shaded with grey/white diagonal hatching (*espB*, *espA*, *sepZ*). Uncharacterized genes where there is no homology, and where their gene products do not appear in our cartoon are shaded solid grey. In the cartoon of the LEE-encoded type-III secretion system proteins are shaded in the same colour as their corresponding genes in the map of the gene cluster. Model based on the type III secretion model from KEGG , and additional published data and images [70, 73, 145, 156, 157]. IM, inner membrane; PG, peptidoglycan layer; OM, outer membrane; EM, eukaryotic membrane.

The important human pathogens, enteropathogenic and enterohaemorrhagic *Escherichia coli *(EPEC and EHEC respectively) utilise type-III secretion to subvert eukaryotic signalling pathways by injecting bacterial effector proteins into the host cell cytoplasm [1,9-12]. Within these pathovars, a well-characterised T3SS is responsible for the development of a characteristic attaching-effacing (AE) lesion and for other effects on enterocyte function [9,11-13] (Figure 1). In common with most other T3SSs, this system is encoded by a "pathogenicity island" (in this case termed the "locus of enterocyte effacement" or LEE), which contains virulence genes clustered on the chromosome and acquired en bloc by horizontal gene transfer [13-16]. Some strains of the rarely isolated putative pathogen of humans, *Escherichia albertii *(formerly misidentified as *Hafnia alvei*), and of the mouse pathogen *Citrobacter rodentium*, which causes transmissible murine colonic hyperplasia, have been shown to induce AE lesion formation and to possess the LEE [17-20].

When applied to biological sequences, homology is defined as similarity that arises because of descent from a common ancestor [21]. Assignments of sequence homology have a predictive value, in that sequence homology implies structural homology, and, less certainly, functional similarity. Although homologous sequences can diverge in function, or be recruited to a different physiological role or cellular compartment, the discovery of homology allows functional assignments to be transferred from a protein or gene that has undergone experimental investigation to a protein or gene that has not been studied in the laboratory (albeit tentatively and allowing for various common pitfalls in this process [22]). Any such functional assignment should always be treated as a working hypothesis rather than as an established fact. However, this approach can lead to new predictions about biological function that can then be tested in the laboratory, informing an existing programme of experimental work, or even opening up an entire line of enquiry where none existed before [22,23]. At its most basic, an assertion of homology calls to our attention the "null hypothesis" – that proteins with homologous sequences should behave in similar ways – and, whenever this does *not* appear to be true, provokes the question, why are these proteins behaving differently? Searches for homology between sequences are reliable only when the search program provides statistics that allow one to estimate how likely it is that randomly composed sequences could yield alignment scores at least as high as that obtained between the two sequences in question (an example of such a statistical evaluation is the e value reported by BLAST [24]).

When the complete sequence of the LEE was first reported, an attempt was made to create a standard rational nomenclature based on experimental findings and functional predictions. *E. coli*-secreted proteins were given the generic name Esp (EspA, EspB, EspD) [11]. Components of the secretion apparatus that had obvious homologues in the better-characterized plasmid-encoded Ysc-Yop T3SS of *Yersinia *sp. were given the prefix Esc and the same suffix as their *Yersinia* homologues (EscC, EscD, EscJ, EscN, EscR, EscS, EscT, EscU and EscV). Proteins that apparently lacked *Yersinia* homologues, but appeared to be involved in type III secretion, retained the designation Sep (for "secretion of *E. coli *proteins"): originally SepZ for rOrf9 and SepQ for Orf17, but with the designations SepD for rorf6 and SepL for Orf23 subsequently seeing widespread use. The chaperone for the secretion of EspD was named *cesD *("chaperone for *E. coli *secreted protein D"). The intimin gene was named *eae *(for "*E. coli *attaching and effacing") and the intimin receptor named Tir (for "translocated intimin receptor"). Since these original designations were made, several other LEE-encoded proteins have been characterized and re-named according to these conventions ((EspF, EspG, EspH; CesF, CesAB/CesA, CesD2), while some others have acquired names outside the original nomenclature, based on functional properties (Ler, Map, GrlR, GrlA) [25-33]

The original assertions of homology for the LEE-encoded proteins – and the functional assignments that flowed from them – were based on the results of unsophisticated searches using the gapped BLAST program [11]. However, in the search for distant homologues, a considerable body of evidence now confirms that simple BLAST searches are far outperformed by more advanced, iterative methods such as PSI-BLAST [24,34-39]. In addition, the growth of domain databases such as PFAM [40] and the steady accumulation of new sequence data on type-III secretion systems, especially from genome sequencing [2,23,41], provide a new backdrop against which original claims of homology (or lack of homology) can now be judged. Bearing these facts in mind – and prompted by a recent assertion that "nearly half of the LEE genes have no homologues [33]" – we have therefore undertaken a fresh bioinformatics analysis of the proteins encoded by the LEE, using highly sensitive methods for the detection of homology. Given the recent discoveries of numerous T3SS effectors secreted through the LEE-encoded T3SS [33,42-46], for reasons of space we have opted to restrict our analysis to LEE-encoded components of the secretion and translocation apparatus and soluble cytoplasmic proteins associated therewith (i.e. chaperones and regulators). We will leave homology analyses of effectors (Tir, EspF, EspG, EspH, Map, SepZ [46] encoded within the LEE; potentially many dozens encoded outside the LEE) and functionally related proteins (intimin) for later publications. We conclude that the vast majority of LEE-encoded proteins do indeed possess homologues and that homology data can be used in combination with experimental data to make fresh functional predictions (Figure 1, Table 1).

**Table 1:** Summary of known characteristics of LEE-encoded genes and their associated proteins, with novel homology-based functional predictions. New names as suggested in body text of this article. Summary of effects on gene expression, type-III secretion and virulence in mice drawn from Deng et al [33]. Yeast two-hybrid results drawn from Creasey et al [145]. Predictions drawn from combination of homology data and cited works. T3S: involved in type-III secretion.

**Gene (new name)**	**Effect of deletion mutation on secretion**	**Effect on pedestal formation / virulence**	**Known interactions**	**Homologues**	**Domains**	**Known functions**	**New functional prediction**
***rorf1***	+	+/+	EspD	YjiK	SdiA-regulated domain (NHL repeats)		SdiA-regulated?
***espG***	+	+/+		VirA		Translocated effector	
***ler***	No expression	-/-		H-NS, StpA		Positive regulator	Might form heterodimers with H-NS, StpA
***orf2 (escE)***	-		Orf29	YscE, PscE, SsaE, CV2595, YPO0259		T3S	Interacts with C-terminus of SepL; Functionally important conserved residues can be identified by scrutiny of homologues
***cesAB***	± EspA, EspB	-/-	EspA, EspB	none		Chaperone for EspA and EspB	
***orf4***	-	-/-		YPO0264, SsaK, CV2589		T3S	Functionally important conserved residues can be identified by scrutiny of homologues
***orf5 (escL)***	-	-/-		YscL, FliH		T3S	Binds to and regulates activity of ATPase EscN
***escR***	-	-/-	EscR, EscS, EscU, SepZ EspD	YscR, FliP	PF00813	T3S	
***escS***	-	-/-	EscR, EspD	YscS, FliQ	PF01313	T3S	
***escT***	-	-/-		YscT, FliR	PF01311	T3S	Physically associated in the basal body with EscU in a 1:1 ratio; C-terminus is cytoplasmic [158]
***escU***	-	-/-	EscrR, EscI, EspD	Yscu, FlhB	PF00771	T3S	Physically associated in the basal body with EscT in a 1:1 ratio [158]; cleaved at NPTH motif [159]; regulates substrate specificity of secretion system [160].
***rorf3 (etgA)***	±	±/±	EscI	lytic transglycosylase IagB, IpgF			Lytic transglycosylase needed to open gap in peptidoglycan for assembly of secretion system [58]; potential drug target
***grIR***	+	+/++	GrlR/GrlA	Bongori regulator		Negative regulator	Will work in concert with GrlA
***grIA***	No expression	-/-	GrlR	Bongori regulator, CaiF		Positive regulator	Will work in concert with GrlR
***cesD***	-/EspD	±/-		Other TPR chaperones		Chaperone for EspD	TPR chaperone [115]
***escC***	-	-/-	EscD	YscC, MxiD, InvG		T3S	Interacts with peptidoglycan [161], Dsb-dependent domain missing
***sepD***	-/Translocators	-/-	SepL	None		Switches translocator/effector secretion	Works in concert with SepL
***escJ***	-	-/-		YscJ, PrgK, FliF	PF01514	T3S	EscD and EscJ form ring-like structure in inner membrane [66, 162, 163]
***rorf8 (escI)***	-	-/-	EscU, rORF3	PrgJ, MxiI, YscI		T3S	Forms inner rod within base of needle complex [65, 68-70].
***sepZ***	+	+/±	EscR	None		Translocated effector	
***orf12***	-	-/-		SsaM		T3S	Might play a role in switching translocator/effector secretion
***escN***	-	-/-	CesT, Tir [50]	YscN		T3S ATPAse	Forms hexameric complex
***escV***	-	-/-		LcrD, FlhA	PF00771	T3S	C-terminal cytoplasmic domain might play a role in gene regulation [164, 165].
***orf15***	-	-/-		None		T3S	
***orf16***	±/Translocators	±/±		None		Secretion of translocators	FliK/YscP analogue?
***sepQ (escQ)***	-	-/-		YscQ, FliN	COG1887	T3S	Forms ring within basal body [56]; interacts with EscL [54]
***espH***	+	+/++				Translocated effector	
***cesF***	±/EspF?	+/+				Chaperone for EspF	
***map***	+	+/++	CesT			Translocated effector	
***tir***	+	-/-	CesT Tir			Translocated effector	
***cesT***	±/Tir	-/-	Map, EspF, Tir, CesT	Many		Chaperone for Tir	Possible regulatory role, by analogy with ExsC?
***eae***	+	-/-				Adhesin (intimin)	
***escD***	-	-/-		YscD, PrgH	YscD: FHA and BON domains	T3S	EscD and EscJ form ring-like structure in inner membrane [66, 162, 163]; BON domains mediate binding to phospholipids; FHA domain mediates protein-protein interactions and signalling
***sepL***	-/Translocators	-/-	SepD	YopN, TyeA		Switches translocator/effector secretion	single protein mediating effects of YopN and TyeA; internal interaction between YopN- and TyeA-like moieties; also a translocated effector?
***espA***	+	-/-	CesAB	flagellin		Translocator	Half-flagellin model (see text); dimerizes before polymerizes; lacks D0 and D3 domains
***espD***	+	-/-	EspD, EscRSU, CesD, rORF1, SepZ	YopB etc		Translocator	
***espB***	+	-/-	CesAB	YopD etc		Translocator	
***cesD2***	±/EspD?	+/±		LcrR		Chaperone for EspD	LcrR will bind YopB
***escF***	-	-/-		YscF		Major needle component	
***orf29***	-	-/-	Orf2	YPO0261, SsaI, H, CV2586		T3S	Functionally important conserved residues can be identified by scrutiny of homologues
***espF***	+	+/+				Translocated effector	

## Results and discussion

### The conserved type-III secretion apparatus

Several LEE-encoded proteins have already been assigned Esc designations to reflect their similarities to the Ysc proteins in the Yersinia Ysc-Yop system [47]. BLASTP-based comparisons of the predicted components of basal complex confirms significant near-full-length homology between EscC, EscD, EscJ, EscN, EscR, EscS, EscT, EscU and EscV and the respective Ysc proteins and homologues (Figure 1, Table 1), although, confusingly, YscV is more commonly known as LcrD, and EscD is sometimes termed Pas [1,47,48]. In the cases of EcsN, EscR, EscS, EscT, EscU and EscV, homology clearly extends to components of the flagellar apparatus [1]. These unequivocal homologues suggest that observations on these proteins in other systems can safely be generalised to the LEE-encoded system – as has been confirmed in several recent papers [49,50] – and can be used to frame novel hypotheses about their function (Table 1, Figure 1). However, it is important to remain on guard for minor but, perhaps, important differences between systems: for example, the C-terminal ~100-residue domain from YscC, which is thought to house two DsbA-dependent disulfide bridges is missing from EscC and many other homologues [51].

**Figure 2:**
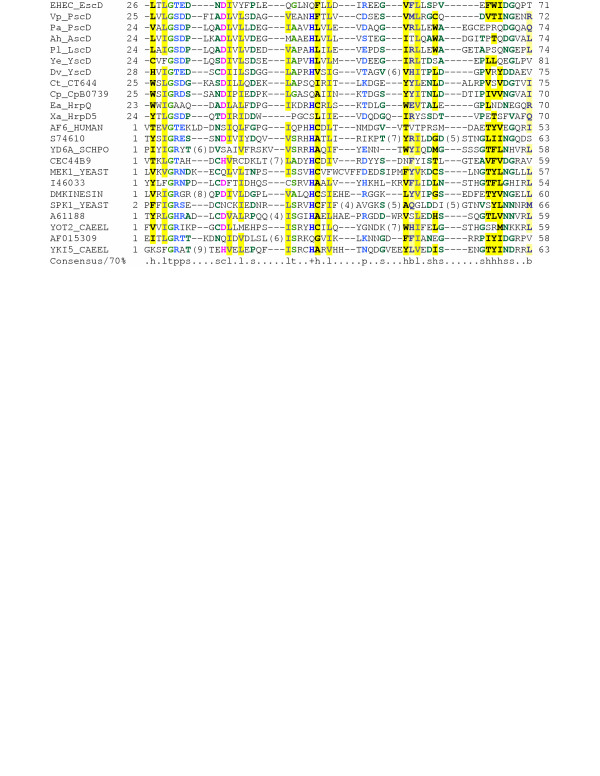
**Multiple alignment of the FHA domain from EscD with other FHA domains. **Alignment is presented using default CHROMA [155] settings: Consensus abbreviations (amino acids): a, aromatic (FHWY, blue lettering on a dark yellow background); b, big (EFHIKLMQRWY, blue on light yellow); h, hydrophobic (ACFGHILMTVWY, black on dark yellow); l, aliphatic (ILV, grey on dark yellow); p, polar (CDEHKNQRST, blue on white); s, small (ACDGNPSTV, dark green on white); t, tiny (AGS, light green on white); -, negatively-charged (DE, red on white); and, +, positively-charged (KR, blue on white), c, charged (DEKRH, pink on white. Organism and gene name abbreviations used: EHEC (*Escherichia coli *O157:H7) EscD (ECs4558/dbj|BAB37981), Vp (*Vibrio parahaemolyticus*) PscD(ref|NP_798074), Pa(*Pseudomonas aeruginosa*) PscD (ref|NP_250408), Ah (*Aeromonas hydrophila*) AscD(gb|AAS91829), Pl (*Photorhabdus luminescens*) LscD(gb|AAO18032), Yp (*Yersinia enterocolitica*) YscD(gb|AAC37021), Dv (*Desulfovibrio vulgaris*) YscD(ref|YP_009153), Ct (*Chlamydia trachomatis*) CT664 (NP_220183), Cp (*Chlamydophila pneumoniae*) CpB0739(gb|AAP98668), Xa (*Xanthomonas axonopodis*) HrpD5 (gb|BAD29996), Ea (*Erwinia amylovora*) HrpQ(gb|AAB06000). The remaining 12 sequences are representative members of the SMART FHA domain (SM00240). Gene names, numbering and alignment are as presented in the SMART FHA family alignment .

**Figure 3:**
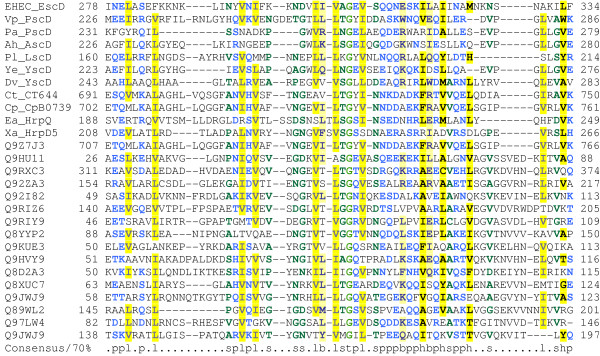
**A multiple alignment of the phospholipid-binding (BON) domain from EscD with other BON domains. **The most obvious BON domain from EscD is shown. However, from patterns of residue conservation centred on conserved glycines in the EscD family of proteins (data not shown), we suspect that there may be one or two more BON-like domains within the cytoplasmic portion of EscD. For sequences 1–11 organism and gene name abbreviations are as for Figure 2 legend. The remaining 16 sequences are representative members of the Pfam BON domain (PF04972). Gene names, numbering and alignment are as presented in the Pfam:BON family alignment . Alignment is presented using default CHROMA settings (see Figure 2 legend).

As noted previously, YscD contains a cytoplasmic FHA domain [52]. PSI-BLAST searches confirm that EscD also contains a cytoplasmic FHA domain (Figure 2). In addition, these searches revealed the presence of at least one putative phospholipid-binding domain (also called a BON domain [53]) in the periplasmic portion of the protein (Figure 3). Two compelling hypotheses arise from these observations. The first of these is that, given the established role of FHA domains in signalling [52], it is tempting to speculate that the cytoplasmic FHA domain of EscD might be involved in signal transduction (alternatively, it might represent a molecular fossil, now fully adapted to a purely structural role). Secondly, as BON domains are thought to mediate binding to phospholipids in a variety of other proteins [53], it is likely that they play a similar role in EscD and its homologues.

### The missing EscL and EscQ proteins

**Figure 4:**
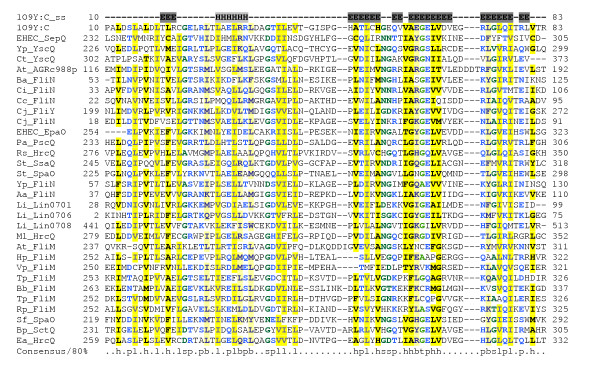
**Multiple alignment of SepQ/EscQ with related type-III secretion proteins. **The sequence and secondary structure of the crystal structure of the C-terminal domain of the HrcQb protein from *Pseudomonas Syringae Pv. Phaseolicola *(pdb|1OY9) are shown 56. Sequences 2–21 (including SepQ) are from COG1886. 1OY9 and the remaining 10 sequences are representative members of Pfam:SpoA. Organism and gene name abbreviations are abbreviated as follows: EHEC (*Escherichia coli *O157:H7) SepQ (ECs4565/gb|AAG58829), Yp (*Yersinia pestis *CO92) YscQ (ref|NP_403921), Ct YscQ (ref|NP_220191), At (*Agrobacterium tumefaciens *str. C58) AGRc988p(ref|NP_353589), Ba (*Buchnera aphidicola*) FliN (sp|P57183), Ci (*Caulobacter vibrioides*) FliN(sp|Q03593), Cc (*Caulobacter crescentus*) FliN(ref|NP_420978), Cj (*Campylobacter jejuni*) FliY (ref|NP_281274) and FliN (ref|NP_281542), EHEC EpaO (ECs3726/sp|Q8X6F0), Pa (*Pseudomonas aeruginosa*) PscQ (ref|NP_250385), Rs (*Ralstonia solanacearum*) HrcQ (ref|NP_522422), St (*Salmonella typhimurium*) SsaQ(sp|P74860) and SpaO (sp|P40699), Yp FliN(ref|NP_404342.1), Aa (*Aquifex aeolicus*) FliN (sp|O67495), Li (*Listeria innocua*) Lin0701 (ref|NP_470044), Lin0706 (ref|NP_470049), and Lin0708 (ref|NP_470051), Ml (*Mesorhizobium loti*) hrcQ (ref|NP_106868), At FliM (sp|Q44457), Hp (*Helicobacter pylori*) FliM (sp|O25675), Bs (*Bacillus subtilis*) FliM (sp|P23453), Bb (*Borrelia burgdorferi*) FliM(sp|57511), Tp (*Treponema pallidum*) FliM (sp|P74927), Vp FliM (sp|Q9Z6GI), Rp (*Rhodobacter sphaeroides*) FliM (O85118), Sf (*Shigella flexneri*) SpaO (sp|P35534), Bp (*Burkholderia pseudomallei*) SctQ (Q9ZGR1), Ea (*Erwinia amylovora*) HrcQ (Q46645). Alignment is presented using default CHROMA settings (see Figure 2 legend).

Although, as noted above, the majority of the "Ysc proteins" that form conserved components of the secretion apparatus in Yersinia have easily recognisable "Esc protein" counterparts in the LEE-encoded system, there are two salient exceptions – in the current annotation of the LEE, there are no equivalents of YscQ and YscL. YscQ is a member of the FliN family of proteins and is thought to be a component of the basal secretion complex [54]. Although PSI-BLAST searches fail to find any YscQ homologue among the LEE-encoded proteins, a search of the NCBI's CDD database [55] identifies a FliN domain (COG1886) in the C-terminal half of SepQ (Figure 4). This suggests that SepQ plays a similar role to YscQ and FliN – allowing new hypotheses to be framed (Table 1) – and should be re-named EscQ. Although, the high degree of divergence of the EscQ C-terminal domain from other YscQ/FliN family members is puzzling, it is clear from scrutiny of the recently solved structure of the homologous domain from HrcQB [56] that SepQ possesses most of the conserved motifs common to this domain family, and must adopt a similar fold (Figure 4).

**Figure 5:**
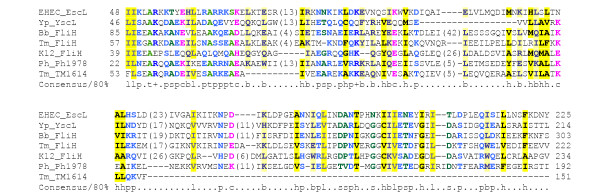
**Multiple alignment of Orf5/EscL with related type-III secretion proteins. **Organism and gene name abbreviations: EHEC (*Escherichia coli *O157:H7) EscL (ECs4584/sp|O85643), Yp(*Yersinia pestis *CO92) YscL(ref|NP_395195), Bb (*Borrelia burgdorferi*) FliH(ref|NP_212423), Tm(*Thermotoga maritim*) FliH(ref|NP_228034), Ph(*Pyrococcus horikoshii *OT3) Ph1978(ref|NP_143803), *E. coli *K-12 FliH (ref|NP_416450), Tm TM1614(ref|NP_229414). Note that Ph1978 and TM1614 are annotated as a hypothetical protein and an ATPase synthase F0, subunit b, respectively. Alignment is presented using default CHROMA settings (see Figure 2 legend).

YscL is a member of the FliH family of proteins. In flagellar systems, FliH binds to and regulates the activity of the ATPase FliI [57], and YscL is also known to bind to YscN [54]. PSI-BLAST searches across the NCBI's non-redundant database with YscL fail to identify any homologue in the LEE system, because of contamination with low-complexity eukaryotic proteins. However, if a PSI-BLAST search with Orf5 is restricted to bacterial proteins, after one iteration, YscL appears in the results list (15% identity 29/184 residues; e value 0.002). After two iterations, several more FliH homologues are found. Furthermore, consistent with the recent suggestion that the YscL-YscN interaction mirrors similar interactions in other ATPases [54], weak similarity is also reported between Orf5 and several F^0 ^ATPase beta subunits (data not shown). A multiple alignment confirms the presence of conserved residues within FliH, YscL and Orf5 (Figure 5). It thus seems likely that Orf5 is a homologue of YscL and FliH, plays a similar role (Table 1, Figure 1) and should be re-named EscL.

### A lytic trans-glycosylase: a novel drug target?

Both domain searches and PSI-BLAST searches show that rOrf3 encodes a lytic transglycosylase. Similar enzymes, which occur in type II, type III and type IV secretion systems, are responsible for enlarging gaps in the peptidoglycan meshwork to allow efficient assembly and anchoring of supramolecular transport complexes in the cell envelope [58]. The presence of a dedicated transglycosylase within this type-III secretion system provides an obvious target for the development of anti-microbial agents that specifically inhibit type-III secretion, particularly as structural data is available for homologous enzymes [59-61]. We propose that rOrf3 be re-named EtgA for *E. coli *transglycosylase. Curiously, we have been unable to find a homologue of EtgA in the Ysc-Yop system.

### The needle

Despite its name, EscF, a major component of the needle, does not show significant similarity to YscF on a simple BLASTP search (19% identity; e value of only 0.88), but the unimpressive significance score can be explained by the short length of the sequence (only 73 residues); the e value rises to a more respectable 0.003 within two iterations of a PSI-BLAST search. Furthermore, it is clear from published experimental work that YscF and EscF play equivalent roles as needle components [62-64].

In the Spi-1 and Mxi-Spa systems, two small proteins are associated with the needle – PrgI/PrgJ and MxiH/MxiI respectively [65-68]. There is experimental evidence from both systems to suggest that only one of the proteins (PrgI/MxiH) is the major subunit component of the needle, while the function of the second protein (PrgJ/MxiI) was, until recently, unclear – it was initially thought to form a cap for the needle [65,68,69]. However, the function of PrgJ (and by extrapolation, of its homologues) has now been elucidated – it forms the inner rod within the base of the needle [70].

**Figure 6:**
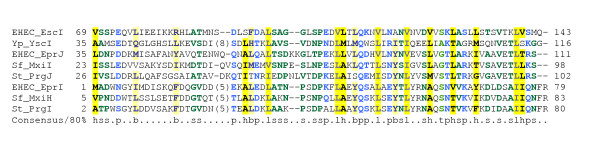
**Multiple alignment of rOrf8/EscI with related type-III secretion proteins. **Organism and gene name abbreviations as follows: EHEC (*Escherichia coli *O157:H7) EscI (ECs4572/sp|O85634) and EprJ (ECs3717/sp|Q8X6G5), Sf (*Shigella flexineri*) MxiI (sp|Q06080) and MxiH (sp|Q06079), St (*Salmonella typhimurium*) PrgJ (sp|P41785) and PrgI (sp|P41784), and Yp (*Yersinia pestis *CO92) YscI (Q00933). Alignment is presented using default CHROMA settings (see Figure 2 legend).

A PrgJ-like component has yet to be described in the LEE-encoded system. However, PSI-BLAST searches indicate that rORF8 is homologous to EprJ, PrgJ, MxiI and to an uncharacterised protein from the Ysc-Yop system, YscI (Figure 6). This suggests that rORF8 and YscI play similar roles to PrgJ/MxiI as components of the inner rod and that rORF8 should be re-named EscI. Interestingly, PSI-BLAST searches also suggest that the PrgI-like and PrgJ-like proteins are paralogous.

### Homology between EspA and Flagellin

**Figure 7:**
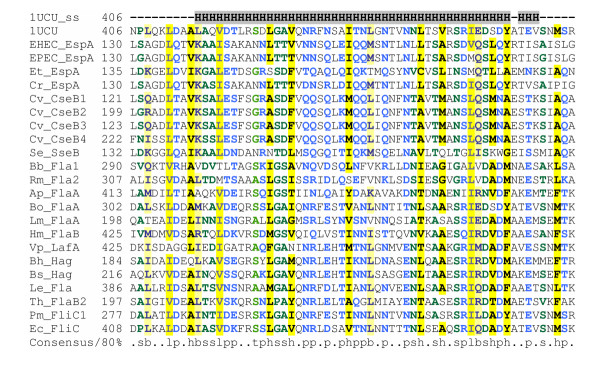
**Multiple alignment of EspA with other EspA-like proteins and flagellins. **The sequence and secondary structure of *S. typhimurium *Sjw1665 R-type straight flagellar filament is shown (pdb|1UCU) [76]. Organism and gene names are abbreviated as follows: EHEC (*Escherichia coli *O157:H7) EspA (dbj|BAB37979), EPEC (*Escherichia coli *E2348/69) EspA (gb|AAC38394), Et (*Edwardsiella tarda*) EspA (gb|AAN52733), Cr (*Citrobacter rodentium*) EspA(gb|AAL06381), Cv (*Chromobacterium violaceum*) CseB1 (gb|AAQ60253), Cv CseB2 (gb|AAQ60252), Cv CseB3 (gb|AAQ60250), Cv CseB4 (gb|AAQ60249), Se (*Salmonella enterica Typhi*) SseB (ref|NP_456130), Bb (*Bartonella bacilliformis*) Fla1 (gb|AAO33576), Rm (*Rhizobium meliloti*) Fla2(gb|AAA26277), Ap (*Aquifex pyrophilus*) FlaA(gb|AAA88923), Bo (*Bordetella bronchiseptica*) FlaA (gb|AAA22977), Lm, (*Listeria moncytogenes*) FlaA (gb|EAL07249), Hm (*Helicobacter mustelae*) FlaB(gb|AAA25017), Vp (*Vibrio parahaemolyticus*) LafA (dbj|BAC62891), Bh (*Bacillus halodurans*) Hag (dbj|BAB07335), Bs (*Bacillus subtilis*) Hag (gb|AAA22437), Le (*Legionella micdadei*) Fla (emb|CAA59172), Th (*Treponema hydodysenteriae*) FlaB2 (emb|CAA45081), Pm (*Proteus mirabilis*) FliC1(gb|AAA62396), *E. coli *K-12 FliC(gb|AAC74990). Alignment is presented using default CHROMA settings (see Figure 2 legend).

The LEE-encoded T3SS is so far unique in possessing a filamentous organelle, the EspA pilus, that resembles the flagellum, forms an extension to the needle complex and is thought to act as a molecular syringe providing a continuous protein-translocation channel between the bacterial and host cells [71-73]. The pilus primarily consists of EspA, but also requires EspD for its biosynthesis [72]. EspB and EspD form a pore in the host cell plasma membrane [74]. PSI-BLAST searches on the ViruloGenome site with EspA reveal homology within two iterations to numerous flagellins, including the *Salmonella* flagellin for which a structure is available [75,76] (Figure 7). Although the precise alignment of the N-terminal region of EspA with flagellin appears critically dependent on the PSI-BLAST settings used, the alignment of the C-terminal region of EspA with flagellin appears robust. This allows certain hypotheses to be framed about the structure of EspA and the EspA filament.

Firstly, surprisingly, the coiled-coil domain at the C-terminus of EspA appears to correspond *not *to the coiled-coil at the extreme C-terminus of flagellin, which forms one half of the flagellin D0 domain, but *instead *to the C-terminal portion of the D1 domain (CD1) [75,76]. In other words, EspA appears to lack a D0 domain. In the flagellar filament, D0 forms the inner tube, while D1 forms the outer tube [75]. Although D0 interactions are important in filament stability, mutant flagellins that lack the D0 domain can still assemble into straight filaments, albeit with a structure, termed Lt, distinct from the native flagellar filaments [77]. It is thus entirely plausible that the EspA pilus is assembled without the need for a D0 domain and resembles the Lt filament structure. One problem with this is that, unlike the native flagellar filament and the EspA filament, the Lt filament appears to lack a central pore. However, this is probably an artefact (it is hard to see how flagellin monomers could be exported without a pore) and by analogy with flagellin [76], one could predict that the central pore in the EspA filament is lined by conserved polar residues close to extreme C-terminus of EspA (e.g. Lys-192).

Secondly, EspA appears to lack a D3 domain (and probably also anything homologous to the D2 domain, although it must contain an analogous surface-exposed domain). This fits in with the observation that D3 is highly variable in size and in sequence among flagellins and is not essential for flagellar filament formation in *Salmonella *[78]. However, it is interesting to note that two EspA homologues from *Chromobacterium violaceum *possess a central insertion, which is likely to fold into an extensive surface-exposed domain, perhaps analogous to the flagellar D3 domain [41].

A third, crucial point concerns the role of the coiled-coil domains in EspA. The coiled-coil domains at the N- and C-termini of flagellin were initially thought to facilitate filament assembly by mediating inter-molecular interactions between neighbouring flagellin subunits [79,80]. Based on the hypothesis that coiled coils might play an analogous role in the assembly of the EspA pilus [72,81], mutagenesis experiments were performed and showed that the C-terminal coiled coil domain of EspA is required for filament assembly [82]. However, the interpretation of these experiments must now be revised in the light of the flagellar structure and homology data. When the flagellar filament structure was solved, it became clear that, contrary to expectation, the coiled-coil domains mediated interactions *within *subunits, rather than *between *them [76]. Thus, by extension, it is likely that, for both EspA and flagellin, the coiled-coil interaction is a necessary but not sufficient requirement for filament assembly.

Coiled-coil prediction programs and PSI-BLAST searches suggest that, in addition to lacking the D0 and D3 domains, EspA lacks any homologue of the N-terminal helix (ND1) of the coiled-coil that forms the D1 domain. However, recent structural information on EspA complexed with its chaperone CesA indicates that EspA contains a short N-terminal coiled-coil domain in addition to the already recognized much longer C-terminal coiled-coil domain [83]. It is thus possible that EspA folds up as a "mini-flagellin", so that together these two coiled-coil domains mediate an intra-subunit interaction similar to that seen in the flagellar monomer. However, this does not fit well with the apparent discrepancy in length between the two domains. An alternative possibility is that EspA acts as "half-a-flagellin", so that it dimerizes through an inter-subunit coiled-coil interaction, and then the dimer polymerizes, like flagellin, through hydrophobic interactions but between dimers rather than monomers.

The "mini-flagellin" and "half-flagellin" models could be distinguished in the laboratory. For example if the half-flagellin model holds true, it should be possible through mutagenesis to identify mutations that disrupt polymerisation but leave the coiled-coil-mediated dimerization potential intact (an approach that might make it possible to grow crystals for structure determination). Furthermore, it should be possible to distinguish dimerization from subsequent polymerisation by physicochemical methods. Finally, under this model, there is a potential for the formation of heterodimers – an idea supported by the existence of four *C. violaceum *EspA homologues, encoded by two pairs of adjacent genes. In each pair, the first gene encodes a short conventional EspA homologue, while the second gene encodes a long EspA homologue containing a central D3-like insertion [41]. According to the half-flagellin model, the short and the long EspA-like proteins would have the potential to form heterodimers, which would then polymerise into a filament. This prediction could be tested experimentally, e.g. by showing that these EspA homologues form long/short dimers but do not engage in self-self interactions.

The Toll-like receptor 5 (TLR5) recognizes the D1 domain of flagellin [84]. Although EspA lacks several of the residues identified as critical in binding (data not shown), the overall conservation of the CD1 domain raises the question of whether TLR5 might recognize EspA. However, recent experimental investigations suggest it does not (K. D. Smith, personal communication). Yet, this in turn raises the question of whether this lack of interaction is the result of genetic drift or of positive selective pressure on EspA to avoid recognition by TLR5.

PSI-BLAST searches with EspB prove unhelpful, as the compositional bias of the protein, particularly in its coiled-coil domains, attracts numerous simple-sequence proteins, even when a filter and composition-based statistics are used [81,85]. However, a comparison of domain architecture and genetic location with translocation-pore proteins/genes from other systems (e.g. SipC, IpaB, YopD) suggests functional and structural similarities. PSI-BLAST searches with EspD prove more fruitful, especially when composition-based statistics are employed – they reveal significant similarities to several other T3SS translocon-pore proteins. For example, when EspD is used as a query sequence for a PSI-BLAST search on the NCBI NR database under default settings, YopB is found in the first round with an e value (0.001) within the PSI-BLAST threshold, while SseC achieves significance (4e-07) on the second PSI-BLAST round.

### Homologies and Gene fusions in the SepL/YopN-TyeA family

**Figure 8:**
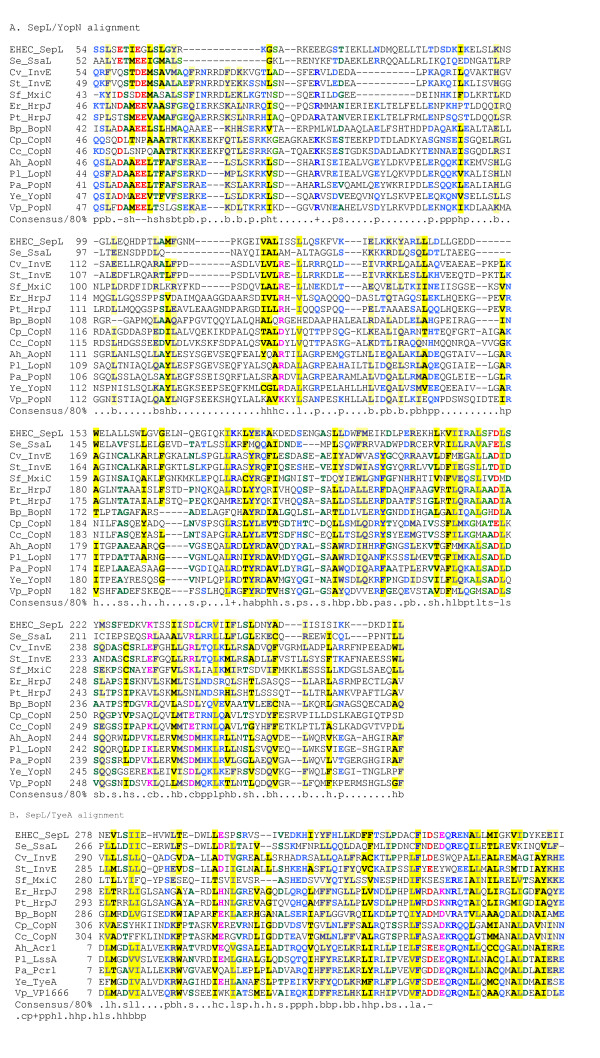
**Multiple alignment of SepL with related type-III secretion proteins, including YopN-like and TyeA-like proteins. **The N-terminal (YopN-like) and C-terminal (TyeA-like) domains are shown separately for clarity. Organism and gene name abbreviations as follows: EHEC(*Escherichia coli *O157:H7) SepL (ECs4557/dbj|BAB37980), Se (*Salmonella enterica Typhi*) SsaL (gb|AAL20336), Cv (*Chromobacterium violaceum*) InvE (gb|AAQ60301), St (*Salmonella typhimurium*) InvE (pir||A46138), Sf (*Shigella flexineri*) MxiC (gb|AAL72332), Er (*Erwinia chrysanthemi*) HrpJ (gb|AAO34609), Pt (*Pectobacterium atrosepticum*) HrpJ (emb|CAD43175), Bp (*Bordetella pertussis*) BopN (emb|CAE42532), Cp (*Chlamydophila pneumoniae*) CopN (gb|AAD18473), Cc (*Chlamydophila caviae*) CopN (gb|AAP05204), Ah (*Aeromonas hydrophila*) AopN(gb|AAR26331) and Acr1(gb|AAR26332), Pl (*Photorhabdus luminescens*) LopN(gb|AAO18045) and LssA(gb|AAO18046), Pa (*Pseudomonas aeruginosa*) PopN(gb|AAC45939) and Pcr1(gb|AAC45940), Ye (*Yersinia enterocolitica*) YopN(gb|AAD16823) and TyeA(gb|AAN37519), Vp (*Vibrio parahaemolyticus*) PopN(dbj|BAC59930) and VP1666(dbj|BAC59929). Alignment is presented using default CHROMA settings (see Figure 2 legend).

SepL is a LEE-encoded protein that has been said to have no homologues and is required for AE lesion formation and secretion of translocation proteins and for the translocation of – but not the secretion of – effectors [86,87]. PSI-BLAST searches reveal homology to two proteins from the Ysc-Yop system: most of the SepL sequence (up to residue 267) is homologous to YopN (sometimes called LcrE), while the C-terminal 83 amino acids are homologous to TyeA (Figure 8). This assertion of homology between SepL and YopN/TyeA is supported by several other lines of evidence (i) the *yopN *and *tyeA *genes are adjacent to one another; (ii) the YopN and TyeA proteins interact with one another; (iii) it is well established that genes encoding interacting domains often occur adjacent to one another and can undergo gene fusion in some genomes, (iv) experimental evidence has recently emerged showing that a chimaeric YopN/TyeA protein can be produced by programmed frame shifting in some Yersinia strains [88-91].

This new-found homology also fuels two predictions: (i) the C-terminal residues 268–351 of SepL, corresponding to TyeA, interact with the residues immediately upstream (229–267) in the region of SepL corresponding to YopN residues 242–280; (ii) the interaction between YopN and TyeA must occur in a 1:1 ratio – this could be confirmed by creating a *yopN*-*tyeA *fusion that lacks a start codon for *tyeA*, and then showing that this construct could complement a *yopN*/*tyeA *mutant to give normal type-III secretion.

Further insights into type-III secretion arise when additional PSI-BLAST searches are applied to the homologues retrieved when SepL is used as the query sequence. Firstly, it is clear that the Ysc-Yop system is unusual in separating out the two SepL-related sequences into two proteins. This arrangement occurs only in systems closely related to the Ysc-Yop system (i.e. T3SSs from *Pseudomonas aeruginosa, Photorhabdus luminescens, Aeromonas salmonicida, Vibrio parahaemolyticus*); in other systems, there is a single protein corresponding to a YopN-TyeA chimaera (e.g. SsaL in Spi-2, HrpJ in *Erwinia *and related species, BopN in *Bordetella*, CopN in *Chlamydia *[92]). Secondly, through careful use of PSI-BLAST, it is possible, through repeated searching, to establish unequivocal homology between SepL-like proteins from each of the well-characterised animal-pathogen-related T3SSs – not just between SepL and YopN-TyeA, but also encompassing InvE from the Spi-1 system and MxiC from the Shigella system (Figure 8). This implies that functional assignments for any one of these proteins should be generalisable to all the others – a prediction borne out by the similar phenotypes ascribed to these proteins, which all appear to play a role in regulating the translocation of effectors [86,87,93-98]. Indeed, it is even possible that some reported differences in phenotype (e.g. the surface location of YopN versus the apparent cytoplasmic location of InvE; the fact that YopN appears to control secretion of all Yops, whereas InvE is more selective in controlling secretion of translocators rather than effectors [98]) might represent idiosyncrasies of experimental approach than genuine biological differences. However, even if these do represent true differences, this begs the question: how and why have homologous proteins come to acquire such divergent functions?

SepL interacts with another protein of unknown function SepD [87]. Unfortunately, homology searches afforded no insights into the function of SepD. However, given that *sepD *is located next to *escC *in the LEE, while *yscB *occupies an analogous position (next to *yscC*) in the *ysc *cluster, it is tempting to speculate that they might play similar roles. Also, no homologues could be found in the LEE-encoded system of LcrV and LcrG, which are thought to regulate entry into the Ysc secretion apparatus from the cytoplasm [99,100]. Similarly, we could not detect any LEE-encoded homologues of the needle-length molecular ruler YscP [101], even though, as we have reported elsewhere [23], PSI-BLAST searches do reveal sequence similarity at the C-terminus between YscP and the flagellar hook-length-determining protein FliK, implying that they employ similar mechanisms [102,103]. Nor could we find any homologue of InvJ/Spa32, which, although showing no detectable sequence similarity to YscP or FliK, is thought to play a similar role to these proteins in needle-length determination in the Spi-1 and Mxi-Spa systems [67,104].

Despite the frustrating lack of sequence similarities, two pieces of tantalising circumstantial evidence link FliK/YscP with InvJ/Spa32, and hint at the identity of an equivalent protein in the LEE-encoded system. Firstly, all four proteins are characterised by the presence of large amounts of random coil on secondary structure predictions (S. Lloyd, personal communication). Secondly, the genes for these proteins always occupy an equivalent position within the type-III secretion gene cluster: always next door but one to and downstream of the gene encoding the ATPase. When these criteria are applied to the LEE, an obvious candidate for needle-length determination emerges: Orf16 (28% random coil according to SOPMA [105]). Almost nothing is known about this gene/protein, which has no known homologues outside of the LEE. However, a mutant in this gene in *Citrobacter *is deficient in translocator secretion, but is still able to secrete effectors normally, and is severely attenuated in the mouse [33]. These results are consistent with the hypothesis that Orf16 controls the switch between export of needle components and components of translocation apparatus. However, in the absence of structural or experimental data, it is premature to make an assertion of homology between Orf16 and YscP/FliK, particularly as tentative assignments tend to harden into dogma and then preclude consideration of other possibilities i.e. that different systems employ different mechanisms for the same purpose. Clearly, additional experimental data are needed to resolve this issue.

### Chaperones

**Figure 9:**
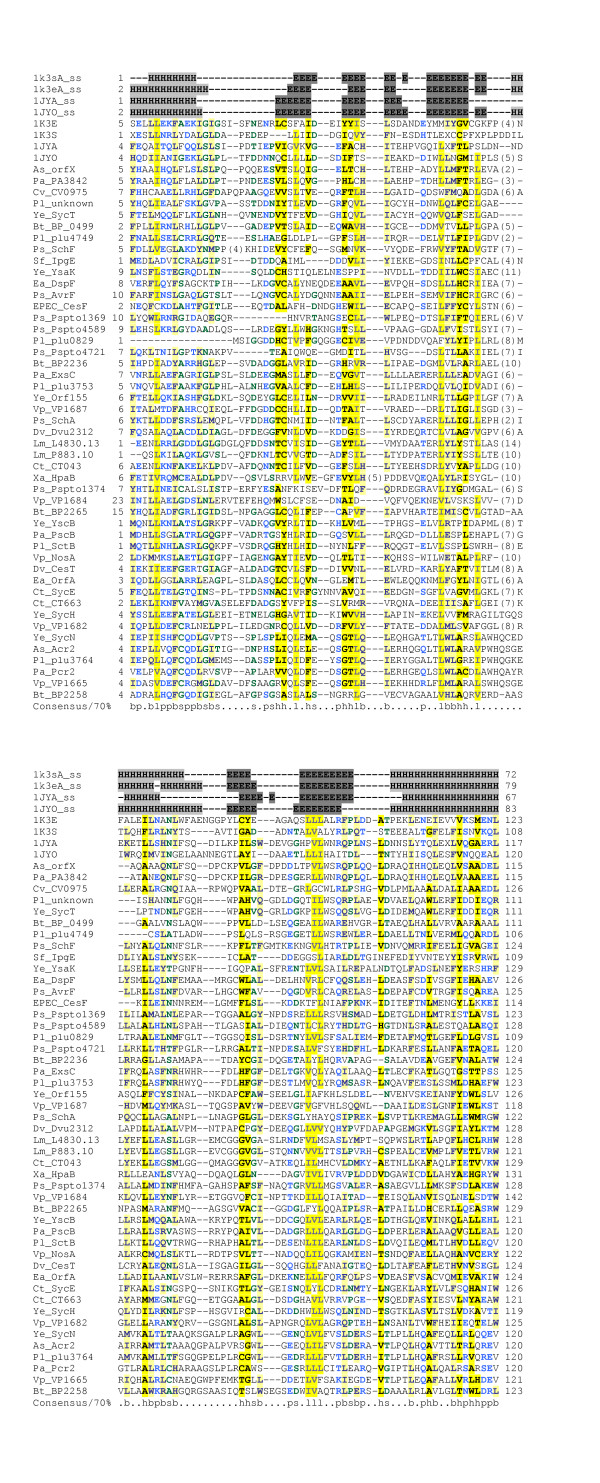
**Multiple alignment of CesT with related type-III secretion chaperones. **Organism and gene name abbreviations as follows: As(*Aeromonas salmonicida*) orfX(gb|AAK83051), Pa(*Pseudomonas aeruginosa*) PA3842(ref|NP_252531), Cv(*Chromobacterium violaceum*) CV0975(ref|NP_900645), Pl(*Photorhabdus luminescens*) unkown(gb|AAO18077), Ye(*Yersinia enterocolitica*) SycT(ref|NP_783658), Bt(*Bordetella pertussis*) BP0499(ref|NP_879351), Pl plu4749(ref|NP_931911), Ps(*Pseudomonas syringae*) SchF(ref|NP_790352), Sf(*Shigella flexineri*) IpgE(gb|AAP78997), Ye YsaK(gb|AAB69191), Ea(*Erwinia amylovora*) DspF(gb|AAC04851), Ps AvrF(ref|NP_791203), EPEC(*Escherichia coli *E2348/69) CesF(gb|AAC38388), Ps Pspto1369(ref|NP_791196) and Pspto4589(ref|NP_794340), Pl plu0829 (ref|NP_928172), Ps Pspto4721(ref|NP_794464), Bt BP2236(ref|NP_880880), Pa ExsC(gb|AAC46214), Pl plu3753(ref|NP_930959), Ye Orf155(ref|NP_783720), Vp VP1687(ref|NP_798066), Ps SchA(ref|NP_795083), Dv Dvu2312(ref|YP_011525), Lm(*Leishmania major*) L4830.13(emb|CAC22615), Lm P883.10(emb|CAC37204), Ct CT043(ref|NP_219546), Xa(*Xanthomonas axonopodis*) HpaB(ref|NP_640751), Ps Pspto1374(ref|NP_791201), Vp(*Vibrio parahaemolyticus*) VP1684(ref|NP_798063), Bt BP2265(ref|NP_880908), Ye YscB(ref|NP_052412), Pa PscB(gb|AAC44773), Pl SctB(ref|NP_930983), Vp NosA(ref|NP_798076), Dv(*Desulfovibrio vulgaris*) CesT(ref|YP_011605), Ea ORFA(gb|AAF63396), Ct(*Chlamydia trachomatis*) SycE(ref|NP_219591), Ct CT663(ref|NP_220182), Ye SycH(ref|NP_863547), Vp VP1682(ref|NP_798060), Ye SycN(ref|NP_052398), As(*Aeromonas salmonicida*) Acr2(emb|CAD30215), Pl plu3764(ref|NP_930970), Pa Pcr2(gb|AAC45941), Vp VP1665(ref|NP_798044), and Bt BP2258(ref|NP_880901). Alignment is presented using default CHROMA settings (see Figure 2 legend).

Five LEE-encoded proteins have been designated chaperones on the basis of experimental work: CesT, CesF, CesD, CesD2 and CesAB [29-31], [106-108]. For one of these, CesT, which chaperones the effectors Tir and Map, the structure is available [109]. PSI-BLAST searches with the CesT sequence link it to CesF and to two other virulence-related T3SS chaperones for which structural information is available (SycE, SicP, SigE [109-112]) and also identify numerous other uncharacterised T3SS-associated proteins as chaperones (Figure 9). Interestingly, the results show homology between CesT/CesF and ExsC from *P. aeruginosa*. As this protein has recently been reported to act as an anti-anti-activator, controlling transcription of T3SS genes in *P. aeruginosa *[113,114], it is plausible that CesT and/or CesF might play similar roles in gene regulation.

CesD is a member of the "chaperones of the translocators" family common to many T3SSs. We have already shown that this family is characterised by the presence of three tandem tetratricopeptide repeats [115]. CesAB (previously Orf3) is a newly described chaperone that binds to EspA and EspB [31]. Homology searches fail to reveal any homologues of this protein, even in the two other systems that possess obvious EspA homologues.

CesD2 (previously Orf27), is a recently described chaperone that, like CesD, binds to EspD [29]. PSI-BLAST searches show that it is homologous to SseE from the Spi-2 system, CseE from the Cpi-2 system of *Chromobacterium violaceum*, LcrR from the Ysc-Yop system, AcrR from *Aeromonas salmonicida *and LssR from *Photorhabdus luminescens*. None of these homologues has been characterised aside from LcrR. An initial report suggested that a mutation in *lcrR *had a regulatory effect [116], although this was subsequently shown to be the result of a polar effect on the downstream gene *lcrG *[117]. However, an *lcrR *mutant was detected in a signature-tagged mutagenesis screen of *Y. pseudotuberculosis *and shown to be attenuated both after oral and intra-peritoneal infection in Balb-C mice, even though the mutation did not have a measurable effect on Yop secretion or function in cell culture assays [118]. These findings stand in contrast to a report that a *Y. pseudotuberculosis lcrR *mutant had no virulence phenotype after oral infection of Swiss albino mice [119]. The homology between LcrR and CesD2 and between EspD and YopB suggests that LcrR is likely to bind to YopB (Table 1).

### Regulators

Ler is a regulatory protein encoded with the LEE1 operon, which has been extensively studied for its effects on gene expression within the LEE-encoded system [120-123]. It is known to be homologous to the global regulator H-NS, and is likely to adopt a similar fold, including a dimeric structure, particularly as key residues in the N-terminal dimerization domain are conserved in both proteins [124-126]. H-NS is known to form heterodimers with other proteins from the H-NS family [124,127,128] and to form complexes with members of the Hha family of regulators [129]. As the attaching and effacing pathogens possess two other H-NS-like proteins besides Ler (H-NS and StpA), and possess Hha-like proteins (Hha, YdgT), it is tempting to ask whether Ler could, under any circumstances, form complexes with H-NS or StpA or with the Hha-like proteins (and if not, why not?).

Quorum sensing through the AI-2 pathway is known to influence type-III secretion in the LEE-encoded system [130-134]. In addition, there is some limited evidence to support an impact on type-III secretion of quorum-sensing through the AI-1/acyl-homoserine lactone route [135]. However, this latter route is less plausible, as *E. coli *cannot produce these signalling molecules, although it can probably sense signals produced by other bacteria through the LuxR-like protein SdiA [136]. It is thus surprising that domain searches with the protein sequence from rOrf1 of the LEE identify a PFAM domain (PF06977) entitled "SdiA-regulated", which represents a conserved region approximately 100 residues long common to YjiK from *E. coli* K-12 and several other bacterial outer-membrane proteins, including some *Salmonella *proteins that are regulated by SdiA [137] (data not shown). This hints at a link between SdiA-mediated sensing of other bacteria and LEE-mediated type-III secretion, which, twinned with growing evidence from *Salmonella *and *Vibrio *for an influence of SdiA/LuxR on virulence and type-III secretion [136,138-141], suggests that it might be worth taking a fresh look at the influence of SdiA on LEE-mediated type-III secretion. Exhaustive PSI-BLAST searches suggest that the "SdiA-regulated domain" in fact consists of several NHL-like repeats (PF01436), which are likely to fold up into a six-bladed beta-propeller [142,143] (data not shown).

GrlR and GlrA (previously known as Orf10/L0044 and Orf11 respectively) are a pair of recently characterised regulatory proteins encoded by adjacent genes within the LEE [33,144]. Similarity has already been reported between GlrA and CaiF, a transcriptional regulator from *E. coli* and to a *Salmonella *protein [33]. PSI-BLAST searches confirm this and report similarity to several other putative transcriptional regulators (e.g. the R721-plasmid-encoded regulator YheC from *E. coli* K-12). PSI-BLAST searches with GlrR identify only one homologue outside the LEE – a protein encoded within the unfinished *Salmonella bongori *genome (data not shown). Interestingly, the *S. bongori glrR *homologue is located next to a *grlA *homologue, suggesting a functional link between the two proteins and adding weight to the finding from yeast two-hybrid studies that GlrA and GlrR interact with each other [145].

### The remaining coding sequences

Six coding sequences, which initially appeared to be orphans, have recently been shown to essential for LEE-encoded type-III secretion: Orf2, Orf4, Orf12, Orf15, Orf16 and Orf29 [33]. Thus, something can now be said about all of them based on homology and/or experimental data. PSI-BLAST searches show that Orf2 has homologues in several other systems: *ssaE *in Spi-2, Cv2595 in Cpi-2 and *yscE *in the Ysc-Yop system (data not shown). Orf2 should thus be renamed EscE. The *ssaE *gene was found to be induced after invasion of a murine cultured cell line [146]. Like YscE in Yersinia [147], Orf2/EscE is necessary for type-III secretion through the LEE-encoded T3SS [33]. YscE interacts with TyeA [148] – thus it would seem likely that Orf2/EscE interacts with the C-terminal portion of SepL (Table 1). YscE also interacts with YscG, a protein with no detectable homologue in the LEE-encoded system [149].

Orf4, Orf12 and Orf29 possess homologues in T3SSs closely related to the LEE-encoded system, although not in the Ysc-Yop system (Table 1). None of the Orf4 or Orf29 homologues has been investigated. However, the homologue of Orf12 in the Spi-2 system, SsaM, has recently been characterised in *S. enterica *[150]. An *ssaM *deletion mutant was attenuated *in vivo *– *in vitro *it over-secreted, but failed to translocate, the effector SseJ. Furthermore, it also failed to secrete translocators. In addition, SsaM was shown to interact with another effector SpiC within the bacterial cell. The story told by this complex set of phenotypes, reminiscent of the multi-faceted YopN-like proteins or of the FliK/YscP-like proteins, stands at odds with the simpler global defect in type-III secretion reported in an *orf12 *deletion mutant [33]. As with the YopN-like and FliK/YscP-like proteins, it remains to be seen whether these apparently dissimilar functional properties of homologous proteins from different T3SSs will converge into a unified mechanistic picture or represent genuine evolutionary divergence of function.

Given that Orf29 is known to interact with Orf2/EscE [145], it is tempting to speculate that, even in absence of homology, Orf29 and the chief binding partner of YscE, YscG might play similar roles. As noted above, Orf16 is a candidate for needle-length control, while the function and evolutionary origin of Orf15 remain mysterious.

## Conclusion

In this survey, we have discovered several new homologous relationships and woven together threads of evidence from our own *in silico *surveys and from published experimental studies to craft numerous novel functional predictions about the LEE-encoded and other T3SSs that can now be tested in the laboratory. We invite the scientific community to test our predictions. Furthermore, in this sequence-rich, post-genomic era [2,23], we believe the time is right to roll out similar bioinformatics analyses over all known type-III secretion systems and invite others to join us in this enterprise on our new web site, 3Base, dedicated to type-III secretion .

## Methods

### Sequences

Peptide sequences of the 44 LEE-encoded proteins from the genome-sequenced enterohaemorrhagic *E. coli *O157:H7 Sakai strain (encoded by genes ECs4548-ECs4591 in the Sakai nomenclature) were extracted from the relevant GenBank file (accession number dbj|BA000007). Analyses were performed on a Macintosh Dual 2 GHz G5 running OSX10.3.

### Homology searches

PSI-BLAST searches [24] were performed on LEE-encoded proteins against a composite database that included the NCBI's non-redundant (NR) protein database and a database of predicted protein sequences from unfinished genomes (VGE-PEPT: searchable on our web site ). To explore all possibilities, multiple PSI-BLAST searches were run for the LEE-encoded proteins using different combinations of substitution matrices (BLOSUM45, BLOSUM62, BLOSUM80), low-complexity filtering (ON, OFF), compositional-based statistics (ON, OFF), E-value inclusion threshold (E = 0.002, E = 0.02) and database (VGE-PEPT + NR, VGE-PEPT alone). PSI-BLAST results were parsed using home-produced Perl scripts and any searches that uncovered significant or near-significant matches to other type III secretion associated proteins were flagged for closer examination and potential homologues retrieved and analysed further. In some cases, PSI-BLAST searches were supplemented with iterative Hmmer searches [151] and searches of the PFAM, CDD and SMART databases [40,55,152].

Most LEE proteins produced statistically significant matches to other Type III associated proteins of known and unknown function under PSI-BLAST default conditions (except for the absence of filtering and compositional based statistics) within 1 to 5 iterations. In some cases (e.g. ECs4584/ORF5), a significant PSI-BLAST hit was dependent on an absence of low-complexity eukaryote sequences in the target database, whereas in others (e.g. ECs4572/rORF8) significant hits were only identified using the BLOSUM80 matrix.

### Alignments

In most cases alignments were generated from homologues identified in PSI-BLAST searches with T-coffee under default settings [153]. In the case of the EscD alignment, the FHA-like and BON-like regions were aligned separately using T-coffee. The former was then aligned to the FHA domain profile (SM00240) from the SMART database and the latter to the BON domain profile from PFAM (PF04972), respectively, using hmmalign from the Hmmer package. Similarly, the SepQ alignment was generated by aligning the COG1886 and Pfam:SpoA domain profiles with hmmalign. The EscI alignment was generated using T-coffee to align homologues parsed from the PSI-BLAST searches before the FliH sequence from *E. coli *K-12 was aligned to it using hmmalign. All multiple alignments were manually curated to ensure that secondary structure elements were not broken and to minimise the number of misaligned regions (as assessed using ClustalX and T-coffee [153,154]). Alignments were coloured using default CHROMA settings [155].

## Authors' contributions

MJP performed the initial PSI-BLAST and CDD searches, managed the project and wrote the paper. SAB performed exhaustive PSI-BLAST searches and other bioinformatics analyses, prepared multiple alignments and compiled these into figures. CMB prepared Figure 1.
